# The Efficacy of High-Dose Dexamethasone vs. Other Treatments for Newly Diagnosed Immune Thrombocytopenia: A Meta-Analysis

**DOI:** 10.3389/fmed.2021.656792

**Published:** 2021-05-25

**Authors:** Qirong Xiao, Bicun Lin, Hanyu Wang, Weiwu Zhan, Ping Chen

**Affiliations:** ^1^Fujian Provincial Key Laboratory on Hematology, Fujian Institute of Hematology, Fujian Medical University Union Hospital, Fuzhou, China; ^2^Central Laboratory, Fujian Medical University Union Hospital, Fuzhou, China

**Keywords:** immune thrombocytopenia, rituximab, dexamethasone, prednisone, clinical trials

## Abstract

**Objective:** To compare the therapeutic efficacies of high dose dexamethasone, prednisone and rituximab in combination with dexamethasone for newly diagnosed ITP (Immune Thrombocytopenia, ITP) patients.

**Methods and results:** Relevant publications for this study were obtained by searching PubMed, Embase, Cochrane, and CNKI (National Knowledge Infrastructure, CNKI) databases following the PRISMA guidelines. A total of, 15 publications were retrieved that contained sufficient data from 1,362 patients for high quality analysis of this study endpoints. Data analysis was carried out using Stata 11.0 software.

The primary outcomes were OR (Overall Response, OR) at 1 month after intervention and SR at 6 and 12 months. The secondary outcomes were AEs and relapse. There were no differences in the OR, while the SR was higher at 6 months (*p* = 0.001) as well as 12 months (*p* < 0.001) in the rituximab + dexamethasone group. In addition, the incidences of AEs (*p* = 0.008) were also higher in the rituximab + dexamethasone group. Dexamethasone was superior to prednisone based on OR (*p* = 0.006). We found no differences in SR at 6 months between dexamethasone and prednisone but SR at 12 months was higher in the dexamethasone group (*p* = 0.014). The relapse rate was higher in the high dose dexamethasone group compared to the rituximab + dexamethasone group (*p* = 0.042).

**Conclusion:** This demonstrated that new treatment options such as Rituximab + dexamethasone, could be a good alternative to traditional therapy in improving long-term response and reducing the rate of relapse. However, further studies are required on the increased risk of AEs associated with Rituximab + dexamethasone.

## Introduction

Immune thrombocytopenia is an autoimmune disease characterized by immune-mediated peripheral platelet destruction. The disequilibrium between the rates of platelet production and destruction in the bone marrow results in varying degrees of risk of bleeding (1). Thrombocytopenia is isolated in primary immune thrombocytopenia patients who may be asymptomatic or have skin and mucous membrane lesions (2). A small number of patients suffer from life-threatening bleeding. A platelet count of <100,000 per cubic millimeter is defined as ITP and a count <30,000 per cubic millimeter is indication for aggressive treatment (3). Different treatments are administered based on the different presentations by patients. Platelet transfusions, glucocorticoids, and intravenous immune globulin are the primary choices for patients who undergo serious bleeding while asymptomatic patients receive treatments depending on platelet count, age, coexisting conditions, and preference (4).

ITP practical guidelines recommend corticosteroid (dexamethasone or prednisone) therapy as the front line therapy. This therapy can effectively reduce the production of antiplatelet and megakaryocyte autoantibodies through immunosuppression and decrease the tendency of bleeding (5). In 2016, Mithoowani reported that there were no significant differences in platelet count response at 6 months in adults treated with high-dose dexamethasone or standard-dose prednisone. This is in contrast to other studies that suggested that high-dose dexamethasone was associated with a high rate of durable platelet count response (6). These contradictory conclusions warrant in-depth discussion and study.

Rituximab, thrombopoietin receptor agonists, splenectomy or cytotoxic drugs are widely used as second-line agents for relapsed and refractory patients. Rituximab is a monoclonal antibody (anti-CD20), which can increase platelet count and response rates. In recent clinical trials, rituximab in combination with corticosteroid or other standard treatments was used as initial treatment and may act as an immunomodulator to enhance the therapeutic effect. However, there is still controversy on the median duration of response and the long-term response rates. A review published in The New England Journal of Medicine indicated that the median duration of response was only 10.5 months when rituximab was used for second line therapy (1). Another meta-analysis (7) published in 2018 reported that rituximab in combination with dexamethasone can improve the long-term sustained response rates at 3–6 months but sustained response rates at later points for 1–3 years was not analyzed. Studies on ITP often failed to evaluate the results of long-term response. There is need to determine if rituximab in combination with dexamethasone could be an alternative to conventional corticosteroid therapy in improving long-term response. The results of a single study are not convincing, hence the need for a meta-analysis study. There is also need to evaluate and compare the therapeutic efficacies of dexamethasone, prednisone, and rituximab in combination with dexamethasone.

## Methods

### Search Strategy and Selection Criteria

We searched PubMed, Embase, Cochrane, and China National Knowledge (CNKI) databases for papers published in English or Chinese, from 2000 to July, 2019. We used the search terms (MeSH): (ITP OR immune thrombocytopenia) AND (randomized controlled trial OR clinical trial) AND (corticosteroids OR prednisone OR dexamethasone) AND (rituximab OR CD20 antibody). The search strategies are outlined in Supplementary Table 1. Two review authors went through titles and abstracts and discarded irrelevant articles. We also searched the key words above for potentially relevant studies.

### Study Selection

Randomized controlled trials, prospective trials and retrospective trials were included in this study.

We selected studies that had included patients that had been newly diagnosed with primary ITP (most had a platelet count <30 × 10^9^/L) in the preceding 3 months and had not received any treatment. Studies eligible for our study had to have a comparator group (either prednisone vs. dexamethasone or dexamethasone vs. rituximab in combination with dexamethasone) and should have reported OR (overall response) rates, SR (sustained response) rates, CR (complete response) rates, PR (partial remission), initial platelet count, or AEs (adverse events). Eligible trials reported Risk ratio (RRs) and 95% confidence intervals (CIs) for overall response and sustained response. We analyzed the most recent data in studies with duplicate publications. A PRISMA flow diagram demonstrated the specific process.

### Data Extraction

Two investigators extracted data from the selected studies in duplicate and then a cross-check for data accuracy was performed. The data collected for each study included basic information such as study name, first author, publication year, gender, age, treatments of enrollment, years of follow-up, and platelet count at diagnosis. In addition, the outcomes assessed were as follows: overall response rates (OR = CR+PR), partial remission rate (PR, platelet count ≥30 × 10^9^/L), complete remission rate (CR, platelet count Data Extraction100 × 10^9^/L), sustained remission (SR, platelet count Data Extraction50 × 10^9^/L) at 6 and 12 months, adverse events (grade 3/4) and relapse.

### Quality Assessment

The Newcastle-Ottawa Scale was used to assess the quality of retrospective studies. The scale has three components, grades studies on the selection of study groups, the comparability of the groups, and the ascertainment of outcome of interest. Studies with scores of <4 were considered to have a high risk of bias, those with scores of 4 to 6 an intermediate risk of bias, and those with scores of 7 or more a low risk of bias (8). The Cochrane Handbook for Systematic Reviews of Interventions was used for randomized controlled trials for risk of bias scaled as high, unclear and low. Any disagreement between the two authors was discussed until a consensus was reached.

### Data Analysis

The results were pooled by using risk ratio (RR) as an effective measurement under the fixed-effect model with 95% confidence intervals. Heterogeneity of studies included was determined using Cochrane *I*^2^ of chi-square-based Q test indices, with a view to further exploring significant heterogeneity (defined as *I*^2^ > 50%) with sensitivity analyses. Funnel plots were appropriate to present. All statistical analyses were carried out using the Stata 12.0 software.

## Results

### Characteristics of Included Studies

**Figure 1:**
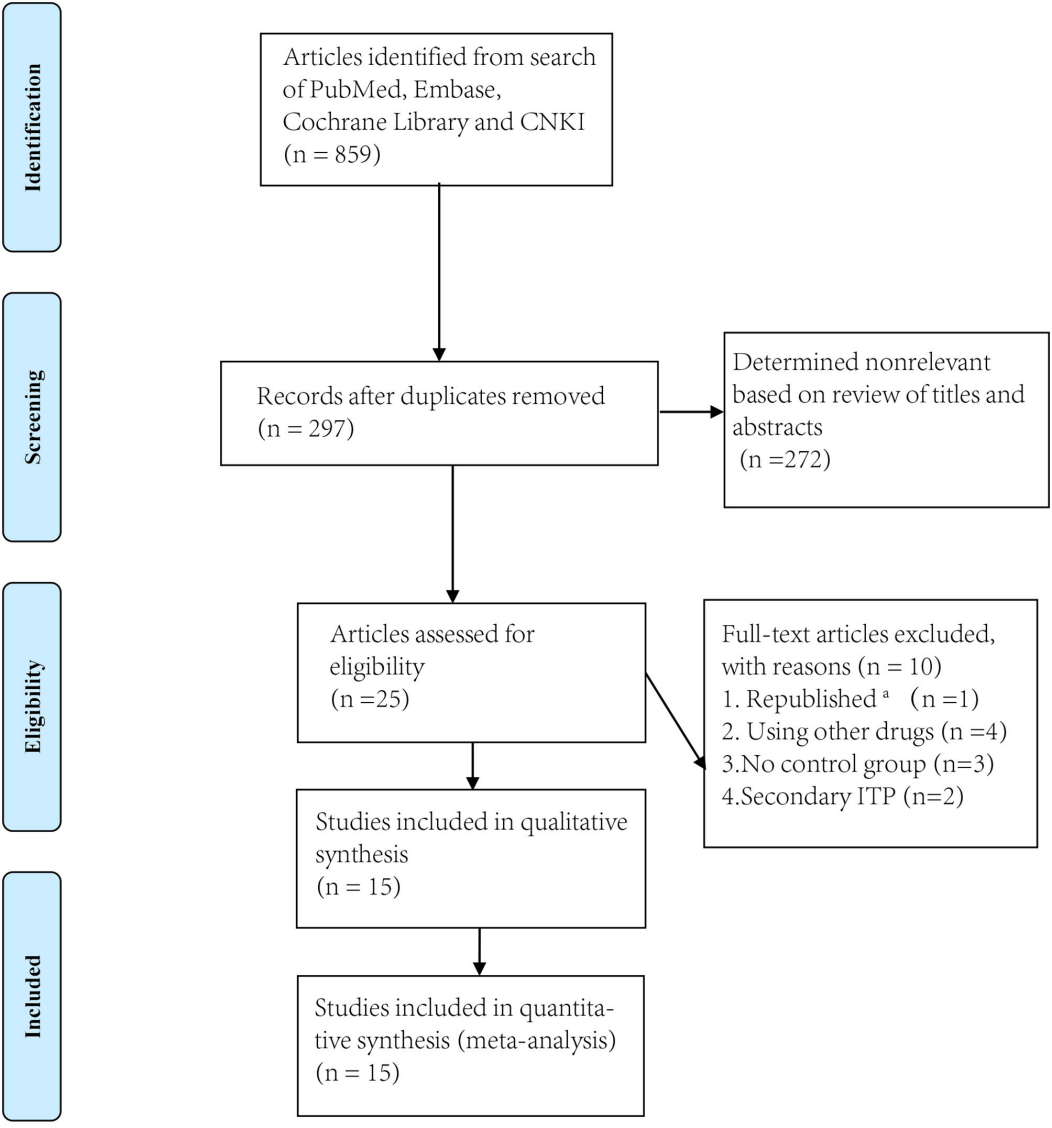
Flowchart of study screening and selection process. ^a^The most updated report was included.

We identified 856 articles from PubMed, Embase, Cochrane Library and CNKI, excluding 272 non-relevant titles and abstracts. We reviewed 351 abstracts and 298 full text articles in duplicate and Independently (Figure 1). Twenty-seven articles published between 2009 and 2019 were assessed for eligibility. Of these, one study was excluded because it had been republished and the most updated report was included. Four studies were excluded because other drugs were used during the treatment. Two studies were excluded because they had no control groups (9, 10).

Thus, ultimately, only fifteen studies met the eligibility criteria (11–25) including 14 RCTs and 1 prospective study for conference (Research and Practice in Thrombosis and Haemostasis) (Table 1). We divided these trials into two arms. Arm A compared dexamethasone with prednisone while arm B compared dexamethasone with rituximab +dexamethasone. Ten trials compared high-dose dexamethasone (40 mg per day for 4 days) with prednisone (0.5–1.5 mg/kg for 2–4 weeks) (11–20) and six trials compared high-dose dexamethasone (40 mg per day for 4 days) with rituximab (either 100 or 375 mg/m^2^ for 4 weeks) combined with high-dose dexamethasone (19, 21–25). Only one trial used a different dose (12.5 ~ 25.0 mg, twice or four times a day) from that of the other nine trials (40 mg daily).

**Table 1:** Summary of included studies.

**References**	**Number of participants (I/C)**	**Age (y), median (range) I C**		**Sex(M/F) I C**		**Disease stage**	**Median duration of follow-up**	**Intervention**	**Regimen (I)**	**Comparison**	**Regimen (C)**	**Initial Plt (×10**^****9****^**/L) median (range) I C**		**Outcomes**	**Relapse**	**Grade 3–4 of AEs**	**6 months**	**12 months**
Din et al. (11)	61/29	29.7 (16–62)	29 (16–64)	27/34	13/16	Newly-Diagnosed	12 months	DXM	40 mg/day^*^4 day	PNS	1 mg/kg ×4 w	1.1 (10–1.9)	1.1 (10–1.8)	CR PR OR SR Aes Relapse	23/13	7/0		
Matschke et al. (12)	13/9	46 (22–77)	43 (29–65)	4/9	5/4	Newly-Diagnosed	12 months	DXM	PNS 1 mg/kg × 1w–> Dex 0.6 mg/kg × 4 d ×6	PNS	1 mg/kg × 2w	2 (0–12)	5 (1–20)		3/7	2/1	11/3	10/2
Praituan and Rojnuckarin (13)	18/18	44.9 (25–64)	39.5 (24–55)	5/13	3/15	Newly-Diagnosed	6 months	DXM	40 mg/day^*^4 day	PNS	1 mg/kg/day^*^28 day	8.5 (0–17.2)	10.3 (1.8–18.8)					
Mashhadi et al. (14)	31/31	24.9 (17–44)	27.2 (18–48)	24/6	23/7	Newly-Diagnosed	12 months	DXM	40 mg/day^*^4 day	PNS	1 mg/kg/day^*^28 day	13.9 (3.4–18)	10.4 (1.5–16.4)		3/10	1/2	27/16	27/14
Sakamoto (15)	31/69	55 (18–86)	61 (18–91)	16/15	30/39	Newly-Diagnosed	12 months	DXM	40 mg/day^*^4day	PNS	0.5–1 mg/kg/day 2–4 w	8	10		17/16	11/14		13/18
Wei et al. (16)	95/97	43 (18–73)	44 (18–75)	64/31	72/25	Newly-Diagnosed	24 months	DXM	40 mg/day^*^4 day	PNS	1 mg/kg/day^*^28 day	7 (0–29)	8 (0–36)		37/32	26/38	40/45	37/32
Bae et al. (17)	76/75	44		20/56	26/49	Newly-Diagnosed	48 months	DXM	40 mg/day^*^4 day	PNS	1 mg/kg/day^*^28 day	16	17					
Cui (18)	30/29	31 (16–62)	34 (18–65)	12/18	10/19	Newly-Diagnosed	3 months	DXM	40 mg/day^*^4 day	PNS	1–1.5 mg/kg × 4 w	10 (0–31)	12 (3–27)					
Li et al. (19)	45/49	37 (19–70)	35 (18–69)	18/27	20/29	Newly-Diagnosed	12 months	DXM	40 mg/day^*^4 day	PNS	1.5 mg/kg × 2–4 w	12 (4–23)	11 (2–19)		23/17			10/17
Nyein et al. (20)	35/35	28.6 (15–42)		30/40		Newly-Diagnosed	6 months	DXM	40mg/day^*^4 day	PNS	1.0 mg/kg BW daily for 4 weeks	ND	ND					
Gomez-Almaguer et al. (21)	20/21	43 (16–83)	51 (18–82)	15/5	17/4	Newly-Diagnosed	33 months	DXM	40 mg/day^*^4 day	DXM + RTX	RTX 100 mg × 4 w –> Dex 40 mg × 4 d	13 (9)	7 (7)		3/10	0/0	17/16	17/16
Gudbrandsdottir et al. (22)	71/62	58 (41–70)	51 (36–63)	37/34	12/36	Newly-Diagnosed	38 months	DXM	40 mg/day^*^4 day	DXM + RTX	RTX 375 mg/m^2^ × 4 w –> Dex 40 mg × 4 d	14 (8–23)	13 (6–20)		41/25	18/27	22/34	17/28
Li et al. (23)	31/31	24 (18–59)	26 (18–51)	12/19	13/18	Newly-Diagnosed	12 months	DXM	40 mg/day^*^4 day	DXM + RTX	RTX 100 mg × 4 w –> Dex 40 mg × 4 d	6 (1–19)	7 (1–17)		12/6	0/0		12/22
Zaja (24)	52/49	47 (28–66)	49 (33–65)	19/33	22/27	Newly-Diagnosed	36 months	DXM	40 mg/day^*^ 4day	DXM + RTX	RTX 375 mg/m^2^ × 4 w –> Dex 40 mg × 4 d	<20	ND		3/7	1/5	19/31	
Li et al. (19)	45/44	37 (19–70)	36 (20–68)	18/27	17/27	Newly-Diagnosed	12 months	DXM	40 mg/day^*^4 day	DXM+RTX	Dex 40 mg × 4 d –> RTX 100 mg × 4 w	12 (4–23)	10 (3–25)		23/13			10/28
Cui (25)	47/48	42 (18–70)	41 (18–70)	19/28	21/27	Newly-Diagnosed	6 months	DXM	12.5 ~ 25 mg, bid ~ qid,1 ~ 4 d	DXM+RTX	RTX100mg, weekly, 28 d –> DXM12.5 ~ 25 mg, bid ~ qid,1 ~ 4 d	18 (15.4–20.6)	18 (15.1–20.9)		22/11			

*RTX, rituximab; DXM, dexamethasone; M, male; F, female; qid, once a day; bid, twice a day; qid four times a day; CR, complete response (platelet count ≥100 × 10^9^/L); PR, partial response (platelet count ≥30 × 10^9^/L); OR, overall response (OR = CR + PR); SR, sustained response (platelet count ≥50 × 10^9^/L at month 6); ND, no determined*.

In arm A, ten trials compared the OR (CR+PR) between the dexamethasone group and the prednisone group at 1 month after intervention. Three trials reported SR at 6 months and five reported SR at 12 months. Six trials compared relapse and five reported grade 3/4 adverse events (such as arthralgia, diarrhea, fever, hyperglycemia, hypertension, infection, insomnia, and mood disorders). In arm B, six trials compared the OR (CR+PR) between the dexamethasone group and the rituximab+ dexamethasone group. Three trials reported SR at 6 months and four reported SR at 12 months. Six trials made the comparison of relapse and four reported grade 3/4 adverse events (such as arthralgia, diarrhea, fever, hyperglycemia, hypertension, infection, insomnia, and mood disorders).

### Quality of Included Studies

The Newcastle-Ottawa Scale and the Cochrane risk of bias tool were used to measure the quality of the included studies. Two retrospective studies and one randomized controlled study were assessed by the Newcastle-Ottawa Scale (Table 2). Two of the cohort trials (15, 21) were assessed as having a low risk of bias. All the studies had a score >5 indicating that all included studies were of good quality (8).

**Table 2:** Newcastle ottawa scale.

**Studies**	**Selection**				**Comparability**	**Outcome**			**Overall**
	**Representativeness of the exposed cohort**	**Selection of the non-exposed cohort**	**Ascertainment of exposure**	**Demonstration that outcome of interest was not present at start of study**		**assessment of outcome**	**Adequacy follow-up length**	**Adequacy of follow up**	
Gomez-Almaguer et al. (21)	1	1	1	0	1	0	1	1	6
Sakamoto et al. (15)	1	1	1	1	1	1	1	1	8

**Figure 2:**
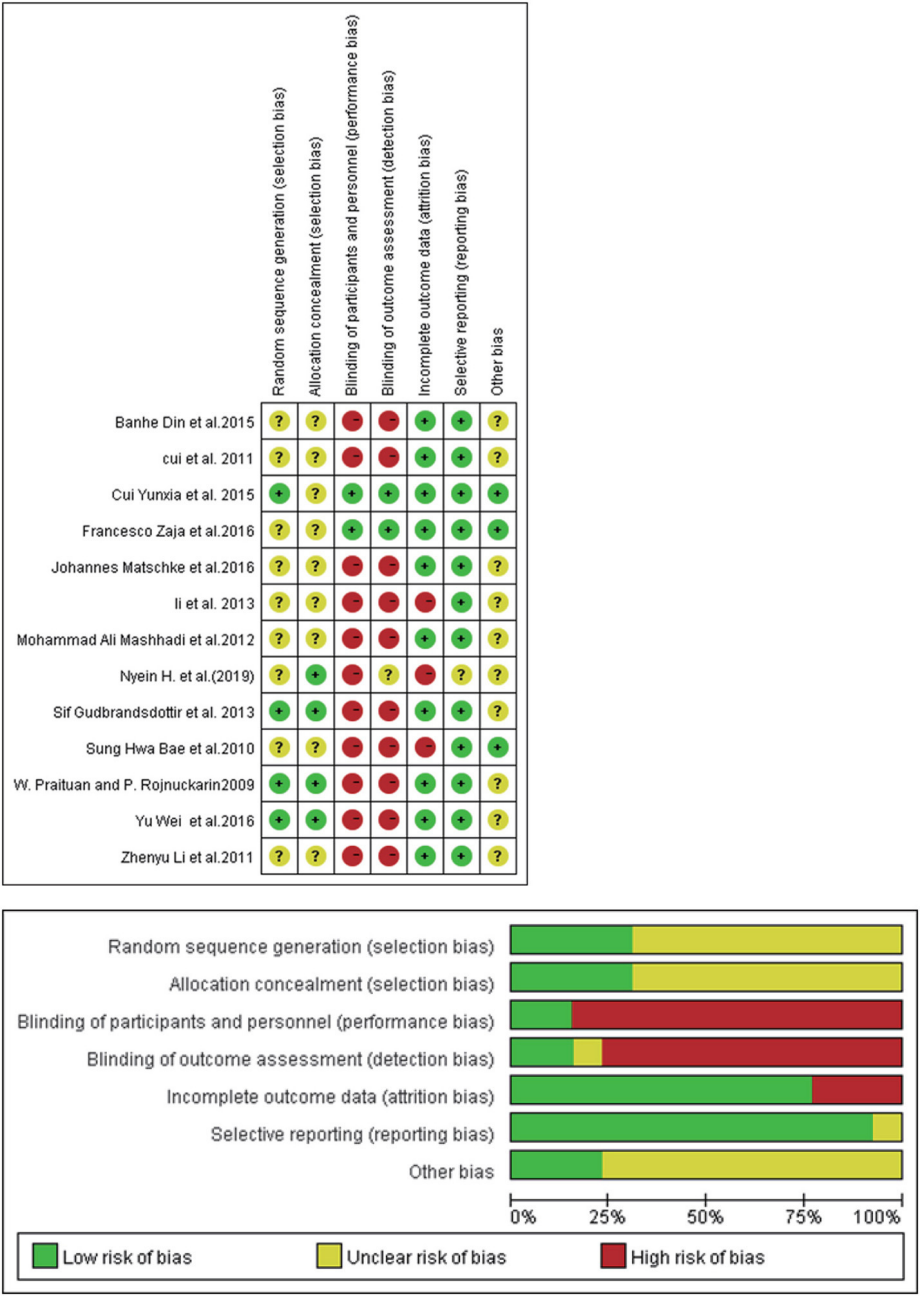
Risk of bias summary: review authors' judgements about each risk of bias item for each included study.

The Cochrane risk of bias tool assessed the quality of randomized controlled trials. The results of the assessment showed that none of the 13 RCTs had high risk of bias, which was evidence that the overall risk of bias was limited (Figure 2).

**Figure 3:**
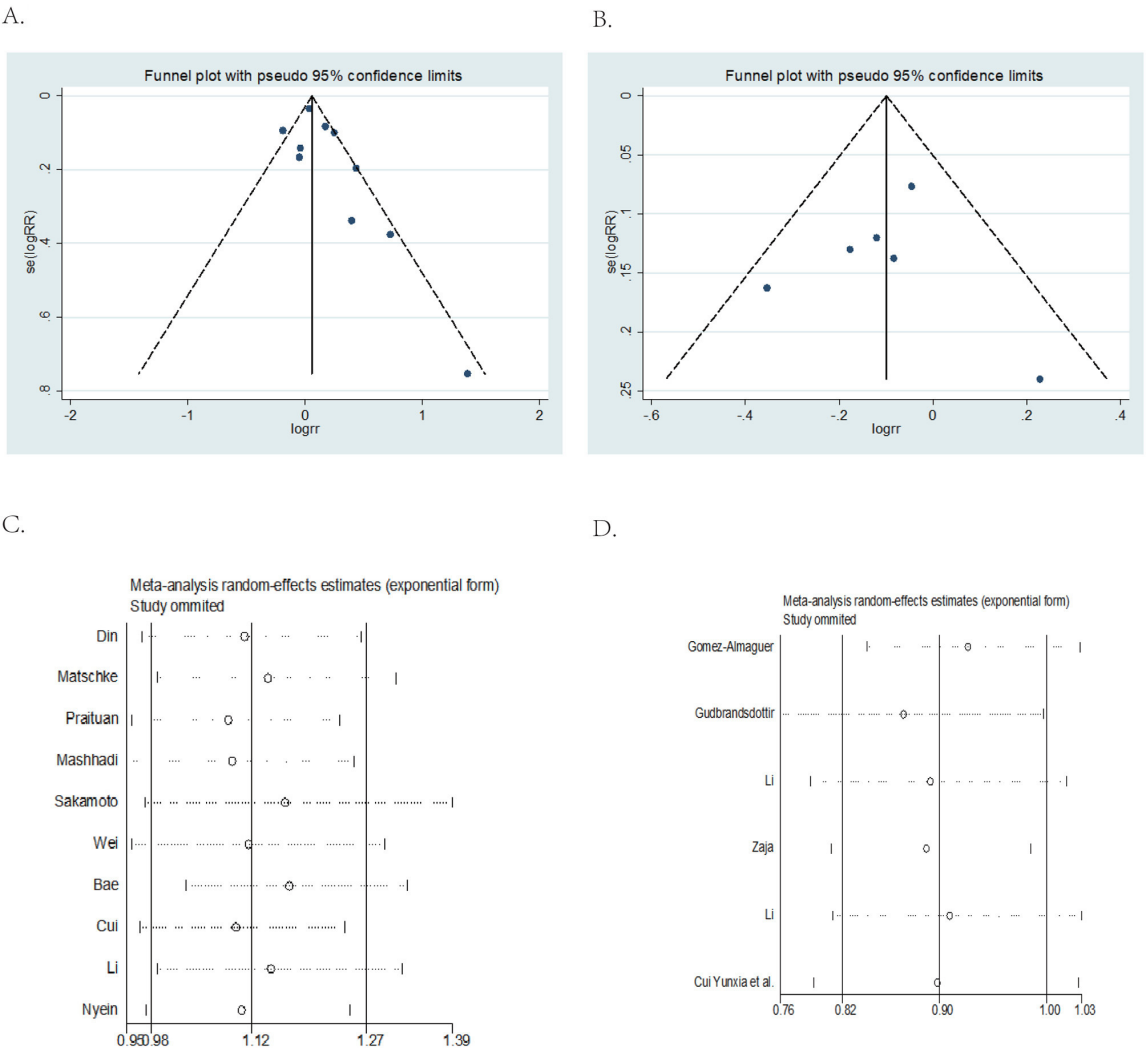
Funnel plot and Egger's test and sensitivity analysis. **(A)** DXM vs. PNS Funnel plot and Egger's test. **(B)** DXM vs. RTX+DXM Funnel plot and Egger's test. **(C)** DXM vs. PNS sensitivity analysis. **(D)** DXM vs. RTX + DXM sensitivity analysis.

Funnel plot analysis for publication bias was performed and presented in Figures 3A,B. Results of funnel plot analysis and Egger's test showed no apparent publication bias (DXM vs. PNS, Egger's test *P* = 0.143 > 0.05; DXM vs. RTX + DXM, Egger's test *P* = 0.873 > 0.05).

Sensitivity analysis showed that omitting any one of the studies separately did not influence the overall result of the pooled analysis (DXM vs. PNS Figure 3C; DXM vs. RTX + DXM Figure 3D).

### Outcomes of Efficacy Analysis

#### Primary Outcomes

##### Arm a (DXM vs. PNS)

**Figure 4:**
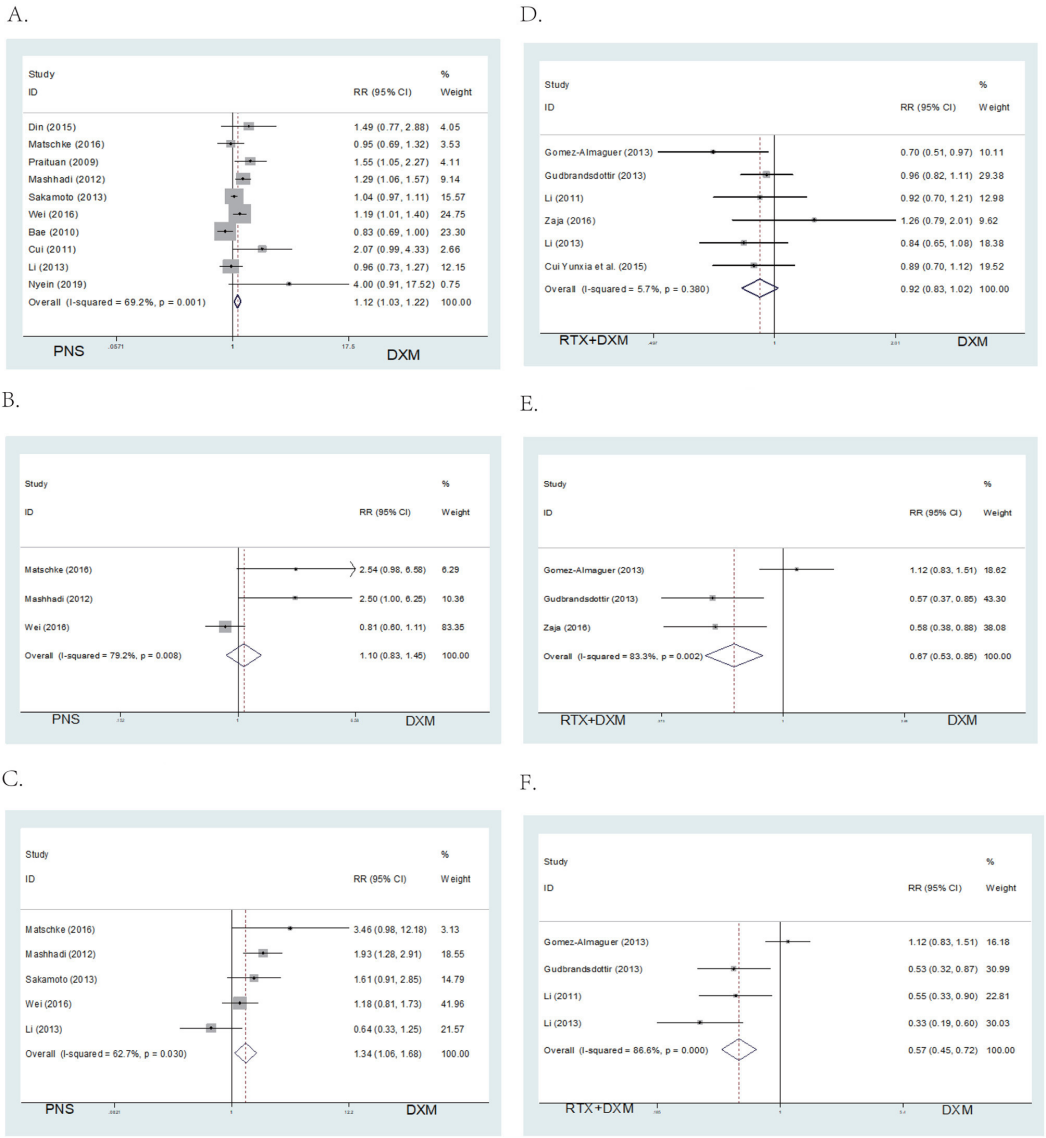
Forest map of OR or SR. **(A)** DXM vs. PNS's OR. **(B)** DXM vs. PNS's SR (6 month). **(C)** DXM vs. PNS's SR (12 month). **(D)** DXM vs. RTX + DXM's OR. **(E)** DXM vs. RTX + DXM's SR (6 month). **(F)** DXM vs. RTX+DXM's SR (12 month).

Data on overall response rates (OR) at 1 month after intervention were available from all ten studies in Arm A. Patients in the high dose dexamethasone group had significantly higher overall response rates (OR) (RR = 1.12; 95% CI = 1.03–1.22, *p* = 0.006, Figure 4A) compared to those in the prednisone group.

There were no differences in sustained response (SR) (RR = 1.10; 95% CI = 0.83–1.45, *p* = 0.517, Figure 4B) at 6 months between the high dose dexamethasone and prednisone groups. However, SR at 12 months was higher in the dexamethasone group compared to the prednisone group (RR = 1.34; 95% CI = 1.06–1.68, *p* = 0.014, Figure 4C).

##### Arm B (DXM vs. RTX ± DXM)

We analyzed the efficacy of high dose dexamethasone compared with rituximab + dexamethasone in newly diagnosed patients. Six trials reported that there was no statistically significant difference in the OR between the high dose dexamethasone group and the rituximab + dexamethasone group at 1 month after treatment (RR = 0.92; 95% CI = 0.83–1.02, *p* = 0.114, Figure 4D). However, in long-term response, the rituximab + dexamethasone group had a higher sustained response (SR) (RR = 0.67; 95% CI = 0.53–0.85, *p* = 0.001, Figure 4E) at 6 months as well as at 12 months of observation (RR = 0.57; 95% CI = 0.45–0.72, *p* < 0.001, Figure 4F).

#### Secondary Outcomes

##### Arm a (DXM vs. PNS)

**Figure 5:**
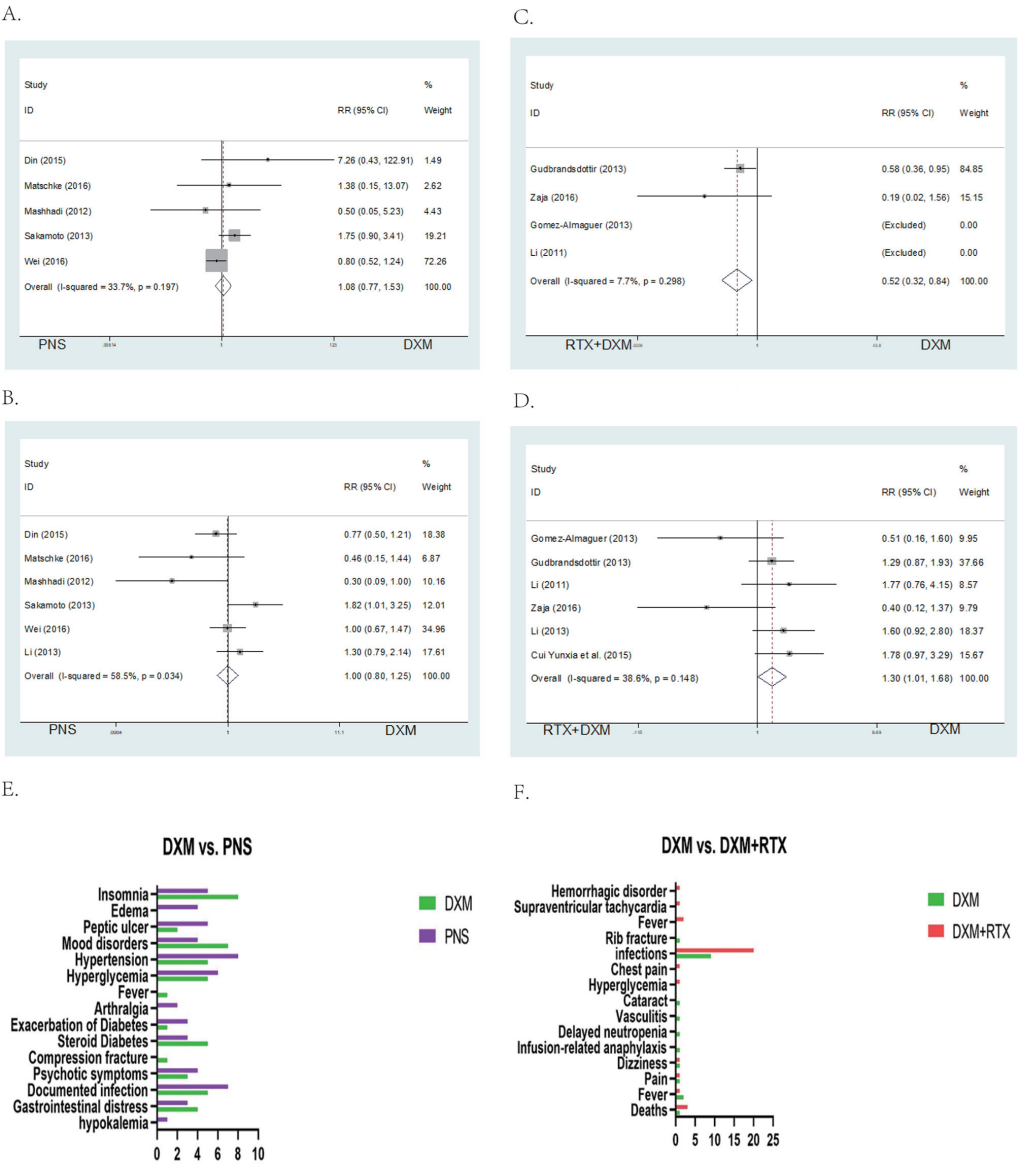
Forest map of AEs or Relapse and bar of AEs. **(A)** DXM vs. PNS's AEs. **(B)** DXM vs. PNS's Relapse. **(C)** DXM vs. RTX + DXM's AEs. **(D)** DXM vs. RTX + DXM's Relapse. **(E)** AEs of DXM vs. PNS. **(F)** AEs of DXM vs. RTX + DXM.

A total of 5 studies provided data for adverse events including arthralgia, diarrhea, fever, hyperglycemia, hypertension, infection, insomnia, and mood disorders (CTCAE grade 3 or 4) (26). Six studies reported the relapse rate. The pooled data showed no significant differences in incidence of adverse events (RR = 1.00; 95% CI = 0.8–1.25, *p* = 0.997, Figure 5A) and relapse rates (RR = 1.00; 95% CI = 0.77–1.53, *p* = 0.648, Figure 5B) in arm A.

##### Arm B (DXM vs. RTX ± DXM)

In arm B, data for adverse events (CTCAE grade 3 or 4, including arthralgia, diarrhea, fever, hyperglycemia, hypertension, infection, insomnia, and mood disorders) were extracted from four studies. The incidences of adverse events (RR = 1; 95% CI = 0.32–0.84, *p* = 0.008, Figure 5C) were higher in the rituximab + dexamethasone group compared to the high dose dexamethasone group. Pooled six studies resulted in significant difference in relapse (RR = 1; 95% CI = 1.01–1.68, *p* = 0.042, Figure 5D), patients in rituximab + dexamethasone group did not result in lower relapse than that in high dose dexamethasone group.

## Discussion

It is essential for ITP patients to rapidly achieve a therapeutic response and maintain long-term responses (27). Thus, we compared the efficacy and safety of existing treatment options which were most commonly used in clinical practice as recommended by ASH guidelines (28).

### In Arm A

We compared high dose dexamethasone with prednisone. Our results showed that high dose dexamethasone had better OR (*p* < 0.05) compared to prednisone at 1 month after intervention. The results of OR were in accordance to a previous study published in 2016 (6). The study also found that there were no differences in the overall platelet count response at 6 months but the overall platelet count response was higher with dexamethasone at 14 days. The in-depth analysis by Siraj confirms our results. In contrast, we selected the first month as the time point for determining overall response after intervention. This was because the pooled results observed different time points for determining OR ranging from half a month to 1 month and we considered that a 1 month observation period could cover all the study outcomes. We included 10 trials in determining OR at 1 month while the previous study only included 4 trials in determining OR at 6 months and 5 trials at 14 days. Both studies had a high risk of heterogeneity (*I*^2^ > 50%). Therefore, we performed the sensitivity analysis (Figure 3C) and none of the studies included showed publication bias. In order to further r, we removed two trials which were abstract-only publications and the result of OR remained unchanged (RR = 1; 95% CI = 1.08–1.30, *p* = 0.001) (Supplementary Figure 1). We therefore concluded that overall response was higher with dexamethasone group than prednisone.

As for long-term responses, there was no difference in SR at 6 months (*p* > 0.05) between high dose dexamethasone and prednisone. However, the SR at 12 months (*p* < 0.05) was higher with the dexamethasone group. These findings were similar to those obtained from studies by Mithoowani et al. (6), confirming that high dose dexamethasone tends to produce a long-term response.

We analyzed the grade 3 or 4 adverse events based on CTCAE even in cases where the events were of low intensity. Results of the analysis showed that peptic ulcer, hypertension, hyperglycemia, diabetes, psychotic symptoms, and documented infection may be decreased in the dexamethasone treatment group. In our statistical analysis, there were no significant differences observed in adverse events and relapse rates between high-dose dexamethasone and prednisone (*P* > 0.05). But Siraj' study reported less frequently with high-dose dexamethasone group. Dexamethasone and prednisone are both corticosteroids with potent anti-inflammatory and immunosuppressive properties. Consequently, their adverse reactions might be similar (Figures 5E,F) (29).

### In Arm B

Our study provided new evidence that treatment with rituximab + dexamethasone did not give statistically significant OR (RR = 0.92; 95% CI = 0.83–1.02, *p* = 0.114) at 1 month after intervention. Interestingly, this finding was in contrast to observations made in previous studies. In 2016, a meta-analysis published by Wang et al. (7) concluded that the efficacy of rituximab + dexamethasone treatment was better as shown by the OR rate at 3 months. In another study by Arai et al. (30) the rituximab + dexamethasone group had a higher OR within 2–4 weeks after the initial therapies. Compared to the study by Arai et al. (30) we included the same four articles (19, 22–24) and added another two trials to our study. This could explain the contradicting findings obtained from the two studies. A systematic review (1) reported that the only second-line treatment that produced sustained increase in the platelet count was splenectomy indicating that the use of rituximab would not lead to a more rapid response.

In the study by Jia Wang, they analyzed six studies and the heterogeneity was high (*I*^2^ =65%> 50%). However, the heterogeneity was much lower (*I*^2^ = 5.7%) in our study. Publication bias may account for the difference in results between the two studies. Thus, more clinical studies are needed to explain the contradictory results.

Our outcomes also provided compelling evidence for long-term response. SR was higher at 6 months (*p* < 0.05) as well as at 12 months (*p* < 0.05) in rituximab + dexamethasone group which was in agreement with a previous study (30). Their network meta-analysis proved that rituximab in combination with dexamethasone can improve the long-term sustained response rate at 3–6 months and this result was confirmed by our work. On that basis, we extended the later points for 12 months. We analyzed the sustain response at 12 months in our Arm B, and the results showed that SR was higher at 12 months (*p* < 0.001) in the rituximab + dexamethasone group. It appears that rituximab + dexamethasone has the capacity to improve the long-term response in ITP patients compared with high dose dexamethasone. In our study, the relapse rate was higher in the high dose dexamethasone group compared to the rituximab + dexamethasone group (*p* = 0.042). Therefore, the choice of rituximab + dexamethasone can improve long-term response and reduce the relapse rates. However, the incidences of adverse events (*p* = 0.008) were higher in the rituximab + dexamethasone group especially in infection. Thus, patient tolerance should be taken into consideration before adding rituximab to the dexamethasone regimen (29).

The limitations of our meta-analysis are listed below: (a) There are various options for second-line treatment such as thrombopoietin receptor agonist, splenectomy, Fostamatinib and so on. But we only compared rituximab + dexamethasone to corticosteroids because other treatments lack evidence from clinical trials. More clinical trials need to be conducted. (b) Two trials were abstract-only publications. Though we made sensitivity analysis and quality assessment, they still run the risk of publication bias. (c) Subgroup analysis was not performed in our study because the sample size was relatively small. (d) The little data on adverse events collected made the certain outcome indicators limited.

## Conclusion

This meta-analysis dem onstrated that high dose dexamethasone was superior to prednisone. Compared to the front-line standard therapy of ITP, rituximab in combination with dexamethasone as a new option for treatment could be a good alternative to traditional therapy in improving long-term response and reducing the relapse rates. However, further studies are required on the increased risk of AEs associated with Rituximab + dexamethasone.

## Data Availability Statement

The original contributions presented in the study are included in the article/Supplementary Material, further inquiries can be directed to the corresponding author/s.

## Author Contributions

QX, BL, WZ, HW, and PC had full access to all of the data in the study and take responsibility for the integrity of the data and the accuracy of the data analysis. All authors contributed to the writing of the manuscript.

## Conflict of Interest

The authors declare that the research wasconducted in the absence of any commercial or financial relationships that could be construed as a potential conflict of interest.

## Abbreviations

CNKI: China National Knowledge
PRISMA: Preferred Reporting Items for Systematic and Meta-Analyses
ITP: immune thrombocytopenia
RTX: rituximab
DXM: dexamethasone
OR: overall response
SR: sustain response
CR: complete response
PR: partial response
AEs: adverse events
RCTs: randomized controlled trials
CTCAE: common terminology criteria for adverse events.
