# Supplementary figures and images for: The Efficacy of High-Dose Dexamethasone vs. Other Treatments for Newly Diagnosed Immune Thrombocytopenia: A Meta-Analysis
Source: Front Med (Lausanne). 2021 May 25;8:656792. doi: 10.3389/fmed.2021.656792 (PMC8185030; doi:10.3389/fmed.2021.656792)

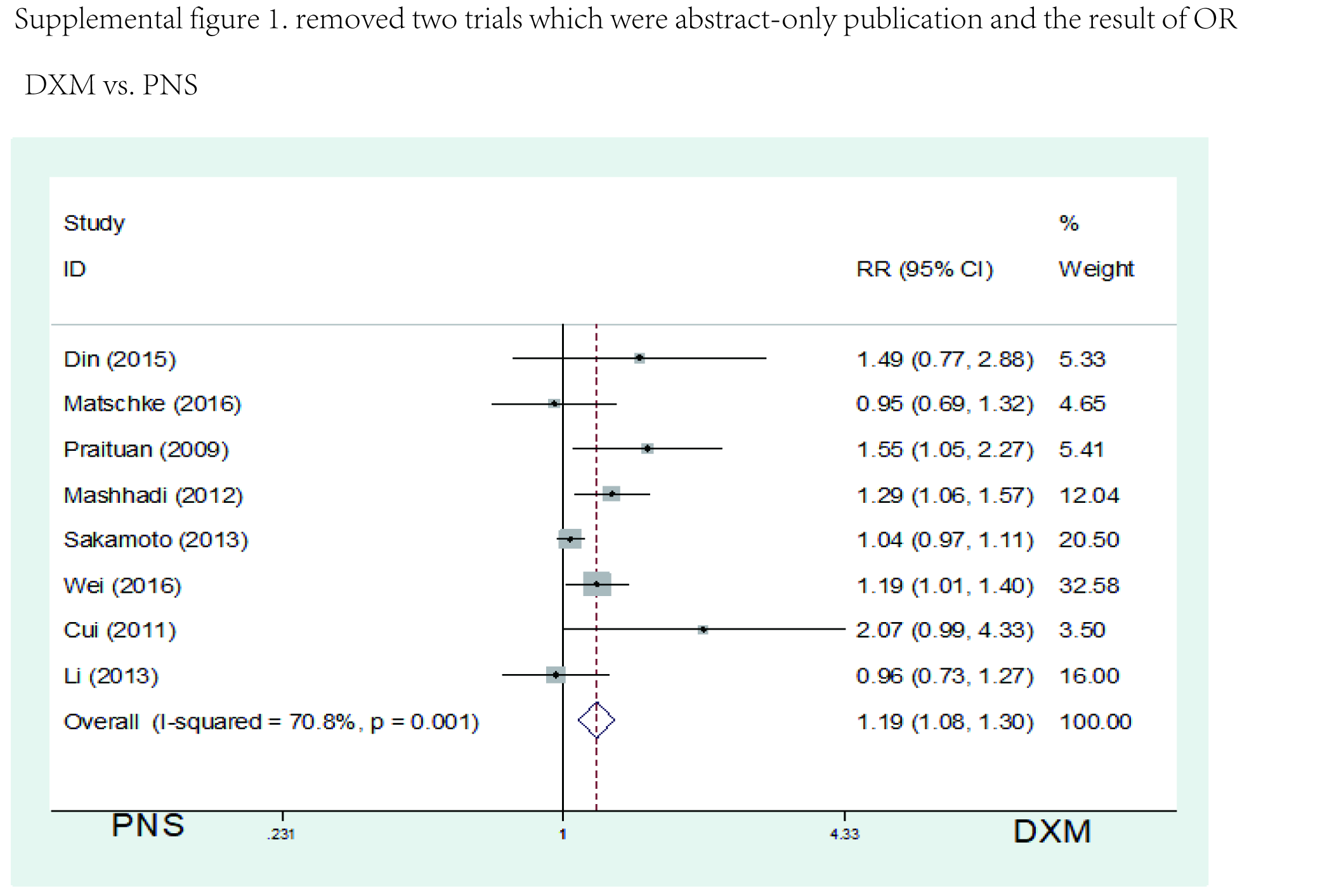

Supplement: Supplementary file 2 [file Image_1.TIF]
